# *N*-(Adamantan-1-yl)-2-chloro­acetamide

**DOI:** 10.1107/S1600536811018046

**Published:** 2011-05-20

**Authors:** Oluseye K. Onajole, Thavendran Govender, Hendrik G. Kruger, Glenn E. M. Maguire

**Affiliations:** a School of Chemistry, University of KwaZulu–Natal, Durban 4000, South Africa; b School of Pharmacy and Pharmacology, University of KwaZulu–Natal, Durban 4000, South Africa

## Abstract

In the title compound, C_12_H_18_ClNO, which was synthesized as part of a study into potential anti­tuberculosis agents, the adamantine skeleton displays shorter than normal C—C bond lengths ranging between 1.5293 (18) and 1.5366 (15) Å. The structure also displays inter­molecular N—H⋯O hydrogen bonding, which forms an infinite chain in the *a*-axis direction.

## Related literature

For background to the title compound, see: Plakhotnik *et al.* (1982 ▶). For the synthesis of the title compound, see: Lee *et al.* (2003 ▶); Bogatcheva *et al.* (2006 ▶, 2010 ▶); Onajole *et al.* (2010 ▶). For related polycyclic structures, see: Venkataramanan *et al.* (2004 ▶); Fokin *et al.*, (2009 ▶).
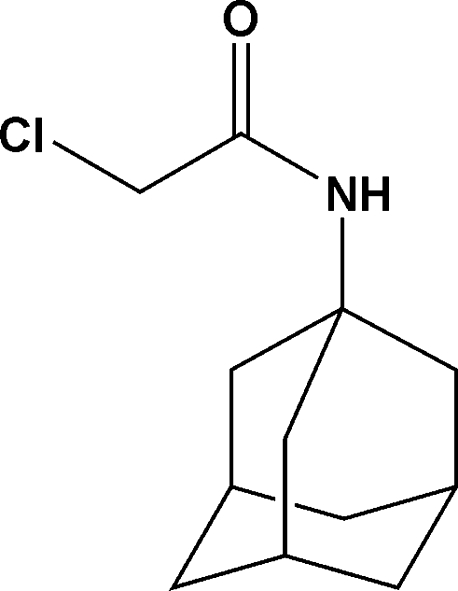


## Experimental

### 

#### Crystal data

C_12_H_18_ClNO
*M* *_r_* = 227.72
Orthorhombic, 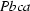

*a* = 9.3656 (2) Å
*b* = 13.7515 (3) Å
*c* = 18.7917 (4) Å
*V* = 2420.20 (9) Å^3^
*Z* = 8
Mo *K*α radiation
μ = 0.29 mm^−1^
*T* = 173 K
0.26 × 0.16 × 0.15 mm

#### Data collection

Nonius KappaCCD diffractometer
Absorption correction: multi-scan (*SADABS*; Sheldrick, 1996 ▶) *T*_min_ = 0.928, *T*_max_ = 0.958
5629 measured reflections
3003 independent reflections
2568 reflections with *I* > 2σ(*I*)
*R*_int_ = 0.007

#### Refinement

*R*[*F* ^2^ > 2σ(*F* ^2^)] = 0.037
*wR*(*F* ^2^) = 0.106
*S* = 1.05
3003 reflections
137 parameters
H-atom parameters constrained
Δρ_max_ = 0.26 e Å^−3^
Δρ_min_ = −0.33 e Å^−3^

### 

Data collection: *COLLECT* (Nonius, 2000 ▶); cell refinement: *DENZO-SMN*; data reduction: *DENZO-SMN* (Otwinowski & Minor, 1997 ▶); program(s) used to solve structure: *SHELXS97* (Sheldrick, 2008 ▶); program(s) used to refine structure: *SHELXL97* (Sheldrick, 2008 ▶); molecular graphics: *OLEX2* (Dolomanov *et al.*, 2009 ▶); software used to prepare material for publication: *SHELXL97*.

## Supplementary Material

Crystal structure: contains datablocks I, global. DOI: 10.1107/S1600536811018046/nk2092sup1.cif

Structure factors: contains datablocks I. DOI: 10.1107/S1600536811018046/nk2092Isup2.hkl

Supplementary material file. DOI: 10.1107/S1600536811018046/nk2092Isup3.cml

Additional supplementary materials:  crystallographic information; 3D view; checkCIF report

## Figures and Tables

**Table 1:** Hydrogen-bond geometry (Å, °)

*D*—H⋯*A*	*D*—H	H⋯*A*	*D*⋯*A*	*D*—H⋯*A*
N1—H1⋯O1^i^	0.88	1.97	2.8301 (12)	165

Symmetry code: (i) 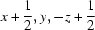.

**Fig. 1.**
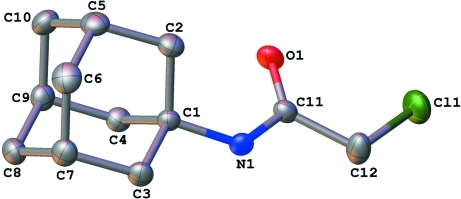
Molecular structure of the title compound with displacement ellipsoids at the 40% probability level and all hydrogen atoms omitted for clarity. All non-hydrogen atoms are shown as ellipsoids with probability level of 40%.

## supplementary crystallographic information

### Comment

As part of an ongoing study into the anti-tuberculosis activity of admantane derivatives (Lee *et al.*, 2003, Bogatcheva *et al.*, 2006, 2010, Onajole *et al.*, 2010), the title compound, an adamantane derivative, serves as a precursor in the synthesis of potential anti-tuberculosis agents (Onajole *et al.* 2010). Although, the compound is known (Plakhotnik *et al.*, 1982), its crystal structure has not been reported.

The molecule displays a number of C—C bond lengths that are shorter than the expected bond length of 1.54 Å. These bonds range between 1.5293 (18) Å for C6—C7 to 1.5366 (16) for C1—C2 in the adamantine skeleton (Fig. 1). The structure exhibits intermolecular hydrogen bonding between N1 and O1 of adjacent molecules, which forms an infinite chain in the *a*-axis direction. The isopropyl (Venkataramanan *et al.*, 2004) amide derivative has a similar bonding arrangement in its structure. Interestingly, the structure report for the bicyclic analogue of the title compund (Fokin *et al.*, 2009) reveals no N–H···O hydrogen bonding in the crystal lattice.

### Experimental

Amantadine.HCl (4 g, 26.5 mmol) was dissolved in dichloromethane (40 ml). To this solution was slowly added chloroacetyl chloride (2.987 g, 26.5 mmol) after which the reaction was refluxed gently for 2 h. The reaction mixture was filtered and the resultant solution was concentrated *in vacuo*. The crude product was purified on silica gel using dichloromethane:ethyl acetate (7:3) as eluent to give the title compound (6.52 g, 89%) as a white crystalline solid. Crystals suitable for X-ray analysis were grown in methanol at room temperature. Melting point: 357–359 K.

### Refinement

X-ray single-crystal intensity data were collected on a Nonius Kappa-CCD diffractometer using graphite monochromated MoKa radiation (l = 0.71073 Å). Temperature was controlled by an Oxford Cryostream cooling system (Oxford Cryostat). The strategy for the data collections was evaluated using the Bruker Nonius "Collect" program (Nonius, 2000). Data were scaled and reduced using *DENZO-SMN* software (Otwinowski & Minor, 1997). Absorption corrections were performed using *SADABS* (Sheldrick, 2008). The structure was solved by direct methods and refined employing full-matrix least-squares with the program *SHELXL97* (Sheldrick, 2008) refining on F^2^. All non-hydrogen atoms were refined anisotropically. All hydrogen atoms were placed in idealized positions in a riding model with *U*_iso_ set at 1.2 times those of their parent atoms and refined with simple bond length constraints (*e.g.* 0.88 Å for N—H and others 0.99 Å).

### Crystal data

**Table:**

C_12_H_18_ClNO	*F*(000) = 976
*M**_r_* = 227.72	*D*_x_ = 1.250 Mg m^−^^3^
Orthorhombic, *P**b**c**a*	Mo *K*α radiation, λ = 0.71073 Å
Hall symbol: -P 2ac 2ab	Cell parameters from 5629 reflections
*a* = 9.3656 (2) Å	θ = 3.0–28.3°
*b* = 13.7515 (3) Å	µ = 0.29 mm^−^^1^
*c* = 18.7917 (4) Å	*T* = 173 K
*V* = 2420.20 (9) Å^3^	Block, colourless
*Z* = 8	0.26 × 0.16 × 0.15 mm

### Data collection

**Table:**

Nonius KappaCCD diffractometer	3003 independent reflections
Radiation source: fine-focus sealed tube	2568 reflections with *I* > 2σ(*I*)
graphite	*R*_int_ = 0.007
1.2° φ scans and ω scans	θ_max_ = 28.3°, θ_min_ = 3.0°
Absorption correction: multi-scan (*SADABS*; Sheldrick, 1996)	*h* = −12→12
*T*_min_ = 0.928, *T*_max_ = 0.958	*k* = −18→18
5629 measured reflections	*l* = −24→25

### Refinement

**Table:**

Refinement on *F*^2^	Secondary atom site location: difference Fourier map
Least-squares matrix: full	Hydrogen site location: inferred from neighbouring sites
*R*[*F*^2^ > 2σ(*F*^2^)] = 0.037	H-atom parameters constrained
*wR*(*F*^2^) = 0.106	*w* = 1/[σ^2^(*F*_o_^2^) + (0.058*P*)^2^ + 0.664*P*] where *P* = (*F*_o_^2^ + 2*F*_c_^2^)/3
*S* = 1.05	(Δ/σ)_max_ = 0.001
3003 reflections	Δρ_max_ = 0.26 e Å^−^^3^
137 parameters	Δρ_min_ = −0.33 e Å^−^^3^
0 restraints	Extinction correction: *SHELXL97* (Sheldrick, 2008), Fc^*^=kFc[1+0.001xFc^2^λ^3^/sin(2θ)]^-1/4^
Primary atom site location: structure-invariant direct methods	Extinction coefficient: 0.0078 (14)

### Special details

**Table:**

Experimental. X-ray single-crystal intensity data were collected on a Nonius Kappa-CCD diffractometer using graphite monochromated MoKa radiation (l = 0.71073?Å). Temperature was controlled by an Oxford Cryostream cooling system (Oxford Cryostat). The strategy for the data collections was evaluated using the Bruker Nonius "Collect" program (Nonius, 2000). Data were scaled and reduced using DENZO-SMN software (Otwinowski & Minor, 1997). Absorption corrections were performed using SADABS (Sheldrick, 2008). The structure was solved by direct methods and refined employing full-matrix least-squares with the program SHELXL97 (Sheldrick, 2008) refining on F2. All non-hydrogen atoms were refined anisotropically. Half sphere of data collected using *COLLECT* strategy (Nonius, 2000). Crystal to detector distance = 30 mm; combination of φ and ω scans of 1.0°, 60 s per °, 2 iterations.^1^H NMR (CDCl_3_, 600 MHz): δ_H_ 1.64 (m, 6H), 1.96 (m, 6H), 2.04 (s, 3H), 3.87 (s, 2H), 6.19 (s, NH).^13^C NMR (CDCl_3_, 100 MHz); δ_C_ 29.3 (CH), 36.1 (CH_2_), 41.1 (CH_2_), 42.8 (CH_2_), 52.3 (C), 164.5 (C=O).
Geometry. All e.s.d.'s (except the e.s.d. in the dihedral angle between two l.s. planes) are estimated using the full covariance matrix. The cell e.s.d.'s are taken into account individually in the estimation of e.s.d.'s in distances, angles and torsion angles; correlations between e.s.d.'s in cell parameters are only used when they are defined by crystal symmetry. An approximate (isotropic) treatment of cell e.s.d.'s is used for estimating e.s.d.'s involving l.s. planes.
Refinement. Refinement of *F*^2^ against ALL reflections. The weighted *R*-factor *wR* and goodness of fit *S* are based on *F*^2^, conventional *R*-factors *R* are based on *F*, with *F* set to zero for negative *F*^2^. The threshold expression of *F*^2^ > σ(*F*^2^) is used only for calculating *R*-factors(gt) *etc*. and is not relevant to the choice of reflections for refinement. *R*-factors based on *F*^2^ are statistically about twice as large as those based on *F*, and *R*- factors based on ALL data will be even larger.

### Fractional atomic coordinates and isotropic or equivalent isotropic displacement parameters (Å^2^)

**Table:**

	*x*	*y*	*z*	*U*_iso_*/*U*_eq_	
Cl1	0.14558 (4)	0.52848 (3)	0.15642 (2)	0.04867 (14)	
O1	0.10158 (8)	0.66702 (7)	0.27321 (5)	0.0342 (2)	
N1	0.33518 (9)	0.70366 (7)	0.29263 (5)	0.0280 (2)	
H1	0.4228	0.6889	0.2797	0.034*	
C1	0.31869 (11)	0.77662 (8)	0.34943 (6)	0.0246 (2)	
C2	0.23383 (13)	0.86513 (9)	0.32293 (6)	0.0316 (3)	
H2A	0.2809	0.8930	0.2804	0.038*	
H2B	0.1361	0.8448	0.3094	0.038*	
C3	0.46993 (11)	0.80966 (9)	0.36968 (6)	0.0304 (3)	
H3A	0.5262	0.7531	0.3863	0.037*	
H3B	0.5182	0.8374	0.3274	0.037*	
C4	0.24600 (12)	0.73351 (8)	0.41519 (6)	0.0278 (2)	
H4A	0.3012	0.6769	0.4325	0.033*	
H4B	0.1488	0.7110	0.4026	0.033*	
C5	0.22636 (14)	0.94200 (9)	0.38192 (7)	0.0360 (3)	
H5	0.1710	0.9995	0.3645	0.043*	
C6	0.37733 (15)	0.97414 (9)	0.40223 (8)	0.0398 (3)	
H6A	0.4255	1.0029	0.3603	0.048*	
H6B	0.3726	1.0242	0.4400	0.048*	
C7	0.46230 (12)	0.88638 (9)	0.42878 (7)	0.0333 (3)	
H7	0.5611	0.9074	0.4418	0.040*	
C8	0.38820 (13)	0.84361 (10)	0.49427 (6)	0.0343 (3)	
H8A	0.3833	0.8931	0.5324	0.041*	
H8B	0.4434	0.7874	0.5123	0.041*	
C9	0.23705 (13)	0.81098 (9)	0.47388 (6)	0.0316 (3)	
H9	0.1886	0.7827	0.5166	0.038*	
C10	0.15142 (13)	0.89847 (10)	0.44707 (7)	0.0369 (3)	
H10A	0.1442	0.9480	0.4851	0.044*	
H10B	0.0536	0.8777	0.4341	0.044*	
C11	0.22906 (11)	0.65799 (8)	0.25909 (6)	0.0275 (2)	
C12	0.28360 (14)	0.59104 (11)	0.20014 (7)	0.0417 (3)	
H12A	0.3506	0.5432	0.2210	0.050*	
H12B	0.3370	0.6302	0.1649	0.050*	

### Atomic displacement parameters (Å^2^)

**Table:**

	*U*^11^	*U*^22^	*U*^33^	*U*^12^	*U*^13^	*U*^23^
Cl1	0.0435 (2)	0.0562 (2)	0.0463 (2)	−0.00949 (15)	−0.01151 (14)	−0.01454 (15)
O1	0.0177 (4)	0.0510 (5)	0.0338 (4)	−0.0016 (3)	−0.0025 (3)	−0.0013 (4)
N1	0.0165 (4)	0.0365 (5)	0.0311 (5)	−0.0006 (3)	0.0008 (3)	−0.0052 (4)
C1	0.0186 (4)	0.0289 (5)	0.0263 (5)	−0.0003 (4)	−0.0003 (4)	−0.0008 (4)
C2	0.0311 (6)	0.0326 (6)	0.0313 (6)	0.0019 (5)	−0.0032 (5)	0.0047 (5)
C3	0.0196 (5)	0.0377 (6)	0.0340 (6)	−0.0036 (4)	−0.0005 (4)	−0.0040 (5)
C4	0.0259 (5)	0.0288 (5)	0.0287 (5)	−0.0026 (4)	0.0014 (4)	0.0022 (4)
C5	0.0370 (6)	0.0284 (5)	0.0425 (7)	0.0061 (5)	−0.0044 (5)	0.0009 (5)
C6	0.0448 (7)	0.0296 (6)	0.0450 (8)	−0.0077 (5)	0.0000 (6)	−0.0006 (5)
C7	0.0251 (5)	0.0375 (6)	0.0371 (6)	−0.0067 (5)	−0.0017 (4)	−0.0062 (5)
C8	0.0338 (6)	0.0390 (6)	0.0302 (6)	0.0002 (5)	−0.0050 (5)	−0.0048 (5)
C9	0.0297 (5)	0.0376 (6)	0.0274 (5)	−0.0026 (5)	0.0039 (4)	−0.0006 (5)
C10	0.0291 (6)	0.0408 (7)	0.0407 (7)	0.0054 (5)	0.0025 (5)	−0.0089 (5)
C11	0.0211 (5)	0.0350 (5)	0.0264 (5)	−0.0015 (4)	−0.0021 (4)	0.0013 (4)
C12	0.0292 (6)	0.0578 (8)	0.0380 (6)	−0.0083 (6)	0.0008 (5)	−0.0170 (6)

### Geometric parameters (Å, °)

**Table:**

Cl1—C12	1.7567 (13)	C5—C10	1.5329 (19)
O1—C11	1.2293 (13)	C5—H5	1.0000
N1—C11	1.3340 (14)	C6—C7	1.5293 (18)
N1—C1	1.4729 (14)	C6—H6A	0.9900
N1—H1	0.8800	C6—H6B	0.9900
C1—C4	1.5304 (15)	C7—C8	1.5303 (17)
C1—C3	1.5354 (14)	C7—H7	1.0000
C1—C2	1.5366 (15)	C8—C9	1.5336 (16)
C2—C5	1.5334 (17)	C8—H8A	0.9900
C2—H2A	0.9900	C8—H8B	0.9900
C2—H2B	0.9900	C9—C10	1.5312 (18)
C3—C7	1.5335 (16)	C9—H9	1.0000
C3—H3A	0.9900	C10—H10A	0.9900
C3—H3B	0.9900	C10—H10B	0.9900
C4—C9	1.5357 (16)	C11—C12	1.5283 (17)
C4—H4A	0.9900	C12—H12A	0.9900
C4—H4B	0.9900	C12—H12B	0.9900
C5—C6	1.5298 (18)		
			
C11—N1—C1	125.80 (9)	C7—C6—H6B	109.8
C11—N1—H1	117.1	C5—C6—H6B	109.8
C1—N1—H1	117.1	H6A—C6—H6B	108.2
N1—C1—C4	111.59 (9)	C6—C7—C8	109.25 (10)
N1—C1—C3	106.53 (9)	C6—C7—C3	109.32 (10)
C4—C1—C3	108.95 (9)	C8—C7—C3	109.82 (10)
N1—C1—C2	111.04 (9)	C6—C7—H7	109.5
C4—C1—C2	109.78 (9)	C8—C7—H7	109.5
C3—C1—C2	108.85 (9)	C3—C7—H7	109.5
C5—C2—C1	109.59 (9)	C7—C8—C9	109.28 (10)
C5—C2—H2A	109.8	C7—C8—H8A	109.8
C1—C2—H2A	109.8	C9—C8—H8A	109.8
C5—C2—H2B	109.8	C7—C8—H8B	109.8
C1—C2—H2B	109.8	C9—C8—H8B	109.8
H2A—C2—H2B	108.2	H8A—C8—H8B	108.3
C7—C3—C1	109.89 (9)	C10—C9—C8	109.62 (10)
C7—C3—H3A	109.7	C10—C9—C4	109.71 (10)
C1—C3—H3A	109.7	C8—C9—C4	109.39 (9)
C7—C3—H3B	109.7	C10—C9—H9	109.4
C1—C3—H3B	109.7	C8—C9—H9	109.4
H3A—C3—H3B	108.2	C4—C9—H9	109.4
C1—C4—C9	109.61 (9)	C9—C10—C5	109.26 (10)
C1—C4—H4A	109.7	C9—C10—H10A	109.8
C9—C4—H4A	109.7	C5—C10—H10A	109.8
C1—C4—H4B	109.7	C9—C10—H10B	109.8
C9—C4—H4B	109.7	C5—C10—H10B	109.8
H4A—C4—H4B	108.2	H10A—C10—H10B	108.3
C6—C5—C10	109.68 (11)	O1—C11—N1	125.04 (11)
C6—C5—C2	109.72 (10)	O1—C11—C12	122.81 (10)
C10—C5—C2	109.21 (10)	N1—C11—C12	112.15 (9)
C6—C5—H5	109.4	C11—C12—Cl1	112.84 (9)
C10—C5—H5	109.4	C11—C12—H12A	109.0
C2—C5—H5	109.4	Cl1—C12—H12A	109.0
C7—C6—C5	109.53 (10)	C11—C12—H12B	109.0
C7—C6—H6A	109.8	Cl1—C12—H12B	109.0
C5—C6—H6A	109.8	H12A—C12—H12B	107.8
			
C11—N1—C1—C4	62.57 (14)	C5—C6—C7—C3	59.83 (13)
C11—N1—C1—C3	−178.63 (11)	C1—C3—C7—C6	−60.28 (13)
C11—N1—C1—C2	−60.26 (14)	C1—C3—C7—C8	59.57 (13)
N1—C1—C2—C5	−176.69 (9)	C6—C7—C8—C9	60.43 (13)
C4—C1—C2—C5	59.45 (12)	C3—C7—C8—C9	−59.46 (13)
C3—C1—C2—C5	−59.73 (12)	C7—C8—C9—C10	−60.35 (13)
N1—C1—C3—C7	179.85 (9)	C7—C8—C9—C4	59.99 (13)
C4—C1—C3—C7	−59.64 (12)	C1—C4—C9—C10	59.49 (12)
C2—C1—C3—C7	60.05 (12)	C1—C4—C9—C8	−60.79 (12)
N1—C1—C4—C9	177.60 (9)	C8—C9—C10—C5	59.74 (13)
C3—C1—C4—C9	60.26 (11)	C4—C9—C10—C5	−60.40 (13)
C2—C1—C4—C9	−58.85 (12)	C6—C5—C10—C9	−59.57 (13)
C1—C2—C5—C6	60.02 (13)	C2—C5—C10—C9	60.70 (13)
C1—C2—C5—C10	−60.23 (13)	C1—N1—C11—O1	−3.61 (19)
C10—C5—C6—C7	60.04 (14)	C1—N1—C11—C12	176.78 (10)
C2—C5—C6—C7	−59.92 (14)	O1—C11—C12—Cl1	−0.73 (17)
C5—C6—C7—C8	−60.38 (13)	N1—C11—C12—Cl1	178.88 (9)

### Hydrogen-bond geometry (Å, °)

**Table:**

*D*—H···*A*	*D*—H	H···*A*	*D*···*A*	*D*—H···*A*
N1—H1···O1^i^	0.88	1.97	2.8301 (12)	165

Symmetry codes: (i) *x*+1/2, *y*, −*z*+1/2.
